# Reductions in cortical alpha activity, enhancements in neural responses and impaired gap detection caused by sodium salicylate in awake guinea pigs

**DOI:** 10.1111/ejn.13474

**Published:** 2016-11-28

**Authors:** Joel I. Berger, Ben Coomber, Mark N. Wallace, Alan R. Palmer

**Affiliations:** ^1^ MRC Institute of Hearing Research University Park Nottingham NG7 2RD UK; ^2^ School of Medicine University of Nottingham Nottingham UK

**Keywords:** behaviour, chronic recordings, electrocorticography, hyperacusis, tinnitus

## Abstract

Tinnitus chronically affects between 10–15% of the population but, despite its prevalence, the underlying mechanisms are still not properly understood. One experimental model involves administration of high doses of sodium salicylate, as this is known to reliably induce tinnitus in both humans and animals. Guinea pigs were implanted with chronic electrocorticography (ECoG) electrode arrays, with silver‐ball electrodes placed on the dura over left and right auditory cortex. Two more electrodes were positioned over the cerebellum to monitor auditory brainstem responses (ABRs). We recorded resting‐state and auditory evoked neural activity from awake animals before and 2 h following salicylate administration (350 mg/kg; i.p.). Large increases in click‐evoked responses (> 100%) were evident across the whole auditory cortex, despite significant reductions in wave I ABR amplitudes (in response to 20 kHz tones), which are indicative of auditory nerve activity. In the same animals, significant decreases in 6–10 Hz spontaneous oscillations (alpha waves) were evident over dorsocaudal auditory cortex. We were also able to demonstrate for the first time that cortical evoked potentials can be inhibited by a preceding gap in background noise [gap‐induced pre‐pulse inhibition (PPI)], in a similar fashion to the gap‐induced inhibition of the acoustic startle reflex that is used as a behavioural test for tinnitus. Furthermore, 2 h following salicylate administration, we observed significant deficits in PPI of cortical responses that were closely aligned with significant deficits in behavioural responses to the same stimuli. Together, these data are suggestive of neural correlates of tinnitus and oversensitivity to sound (hyperacusis).

## Introduction

Sodium salicylate, an analogue of salicylic acid (the active ingredient of aspirin), reliably induces a tinnitus in both humans and animals (for a review, see Stolzberg *et al*., 2012). Tinnitus, defined as the perception of sound in the absence of an external auditory stimulus, is a symptom that is experienced chronically by 10–15% of the population (Baguley *et al*., 2013). The triggering cause in the majority of cases is likely to be hearing loss as a result of noise overexposure. Nonetheless, given the reliability of salicylate in inducing tinnitus compared with the uncertain outcome of noise exposure, it is a useful research tool for understanding neural changes associated with the presence of tinnitus.

Salicylate administration at moderate‐to‐high doses is well known to have reversible ototoxic effects (Stypulkowski, 1990). This is suggested to occur through a reduction of outer hair cell electromotility (e.g. Kakehata & Santos‐Sacchi, 1996). Central changes have also been demonstrated in anaesthetized animals, whereby auditory evoked potentials are enhanced following salicylate administration (e.g. Lobarinas *et al*., 2006; Yang *et al*., 2007). However, Sun *et al*. (2009) indicated that anaesthesia may affect salicylate‐induced changes in neural activity. Given that anaesthesia temporarily abolishes consciousness (Alkire *et al*., 2008) and the presence of tinnitus by definition requires conscious perception, it is important to understand the induction of tinnitus by salicylate in awake animals.

Despite its wide use in animal models of tinnitus, there are relatively few studies that have examined neural activity following salicylate in awake animals (Lu *et al*., 2011; Zhang *et al*., 2011; Stolzberg *et al*., 2013; Sawka & Wei, 2014). Salicylate has been shown to reduce the size of auditory brainstem responses (ABRs; Pienkowski & Ulfendahl, 2011), indicative of auditory processing in the periphery and brainstem, and contrastingly enhance cortical evoked responses (Yang *et al*., 2007; Sun *et al*., 2009; Norena *et al*., 2010). Only two studies have examined oscillatory activity in awake animals following an intervention that causes tinnitus. Norena *et al*. (2010) demonstrated a decrease in activity over a fairly broad frequency range (~ 8–35 Hz) following both salicylate and noise trauma in guinea pigs (GPs). Subsequently, preliminary data from Stolzberg *et al*. (2013) in rats indicated that salicylate caused decreases in delta and alpha band activity, as well as an increase in low gamma activity (~ 20–80 Hz).

Given that changes in oscillatory activity have been proposed as biomarkers for tinnitus in humans (for a review, see Adjamian *et al*., 2009), it is important to further explore these in an animal model. Here, we recorded electrocorticographic (ECoG) activity from awake GPs, before and after the administration of salicylate. In the same animals, we examined resting‐state oscillatory activity, wave I ABRs (which informed us of peripheral activity), gap detection thresholds, which provided a measure of temporal acuity, and click‐evoked potentials.

Furthermore, a commonly used behavioural test for tinnitus in animals is known as gap‐pre‐pulse inhibition of the acoustic startle (GPIAS; Turner *et al*., 2006; Turner & Parrish, 2008; Longenecker & Galazyuk, 2011; Dehmel *et al*., 2012; Turner *et al*., 2012; Berger *et al*., 2013; Chen *et al*., 2013; Coomber *et al*., 2014). This test exploits a phenomenon whereby a response to a startling stimulus can be inhibited by a preceding gap in otherwise continuous background noise. The original hypothesis for the test was that, if the animal is experiencing tinnitus after a particular intervention (such as salicylate administration), the gap will become less salient and the startle response will not be inhibited as effectively. However, two recent studies in humans have cast doubt on this interpretation of behavioural gap detection deficits (Campolo *et al*., 2013; Boyen *et al*., 2015), as well as one study in rats (Radziwon *et al*., 2015) and a review (Eggermont, 2013).

An important aspect of all the studies that have failed to find a difference in gap detection abilities in patients with tinnitus, as well as in salicylate‐administered rats, is that they determined either gap detection thresholds or psychophysical gap detection abilities, rather than inhibiting a startle response with a preceding gap. Moreover, by employing a GPIAS paradigm, Fournier & Hebert (2013) were able to demonstrate significant deficits in gap detection in tinnitus patients, albeit at frequencies below their tinnitus. Indeed, work from our own laboratory indicated that minimum gap detection thresholds (MGDTs) in the inferior colliculus were slightly increased following noise exposure, but not to an extent that could explain behavioural gap detection deficits that were present in the same animals (Berger *et al*., 2014).

Weible *et al*. (2014) recently demonstrated that they could affect the degree of startle attenuation (as measured behaviourally) by using optogenetics to alter inhibitory and excitatory activity in the auditory cortices of mice, while preserving MGDTs. This therefore implies that there may be disparate underlying mechanisms between gap detection thresholds, which they suggested are a measure of temporal acuity, and inhibition of a startle response by a preceding gap, which they proposed as a measure of gap salience. To examine these differences, in a separate group of GPs but with the same dose of salicylate as used for measuring MGDTs, we recorded behavioural GPIAS responses and awake neural responses to the same stimuli as used in the behavioural test, in order to further explore the potential mechanisms behind the GPIAS test. We hypothesized that we would (i) show behavioural gap detection deficits in GPs administered salicylate, (ii) demonstrate MGDTs in auditory cortex were not altered sufficiently to explain such behavioural deficits following the same dose of salicylate, (iii) show for the first time that evoked responses in auditory cortex can be inhibited by a preceding gap in otherwise continuous background noise [termed gap‐induced reductions of evoked responses (GIREP)], and (iv) find deficits in GIREP following salicylate administration that corresponded to behavioural deficits in the same GPs.

## Materials and methods

### Animals

All procedures were carried out in accordance with the European Communities Council Directive of 24 November 1986 (86/609/EEC) and the approval of the Animal Welfare and Ethical Review Body at the University of Nottingham, UK. Experiments were conducted on a total of 13 tricolour GPs (five male and eight female) weighing 500–800 g at the time of implantation.

### ECoG array implantation

To record ECoG signals, a custom electrode array was first prepared. This consisted of eight uninsulated silver wires heated to produce a ball on the end in order to prevent damaging the dura mater when implanted. These were soldered on to a circuit board attached to a Tucker Davis Technologies (TDT, Alachua, FL, USA) zero insertion force (ZIF)‐clip connector.

During implantation, which was performed aseptically, GPs were initially anaesthestized with ketamine (40 mg/kg, i.p.) and xylazine (8 mg/kg, i.p.) and then artificially respired on an isoflurane/O_2_ mixture throughout the procedure to maintain a constant state of areflexia. Core body temperature was maintained at 38 ± 0.5 °C using a homeothermic heating pad (Harvard Apparatus Ltd., Edenbridge, UK) and a rectal probe. The head was shaved and wiped with iodine. Lidocaine injections were administered subcutaneously around the area of incision. An incision was made along the midline of the head, from ~ 4 mm in front of bregma to the nuchal ridge, as well as small lateral incisions (~ 3 mm) at either end. Muscle and connective tissue were cleared and small burr holes for electrode placement were made in the locations described below. Two small screws were inserted into the skull to act as anchoring points for the electrode array.

**Figure 1:**
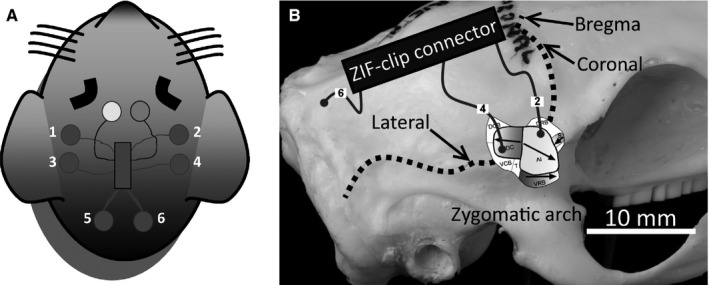
(A) Illustration of GP head with silver‐ball electrodes positioned over left and right rostral AC (1–2), caudal AC (3–4) and cerebellum (5–6), as well as linked reference and ground electrodes (white and grey front circles). (B) Positioning of AC electrodes with respect to skull landmarks and the different areas of the auditory cortex, based on a reconstruction from Grimsley *et al*. (2012).

A schematic of the electrode positions is shown in Fig. 1A. Individual electrodes were placed into burr holes over rostral and caudal AC on each side, while a further two were positioned over cerebellum on each side in order to record ABRs. Reference and ground electrodes were linked via a jumper on the electrode board and implanted ~ 3 mm rostral to bregma, just off midline on either side. Based on coordinates from Grimsley *et al*. (2012), the rostral and caudal electrodes were putatively over the dorsorostral edge of primary AC (abbreviated to rostral) and the dorsocaudal area (abbreviated to caudal) respectively (see Fig. 1B).

After electrode insertion, burr holes and the underside of the electrode board were first covered with Kwik‐Cast silicone sealant (World Precision Instruments, Hitchin, UK) and then with dental acrylic. The wound was sutured with Mersilk (Ethicon, Livingston, UK) and covered in antibiotic cream. Cyanoacrylate was applied to stick the skin around the board. GPs were removed from the isoflurane and placed in a recovery box until they were stable enough to return to their home cage. GPs were left for at least 24 h before beginning baseline ABR and ECoG recording.

### Recording setup

For chronic recording of ABR and ECoG signals, a custom‐made cage (30 × 15 × 21 cm) surrounded by acoustic foam was placed inside a sound‐attenuating chamber. An infrared webcam was placed above the cage to monitor the animal's movement. Animals were not restrained and were allowed to move freely within the cage. Auditory stimuli were presented free‐field via a single ¾‐inch tweeter (Tymphany XT19TD00) positioned ~ 30 cm above the centre of the cage. Sound pressure level calibration was performed before each recording session using custom‐written Matlab scripts and two ¼‐inch free‐field microphones (G.R.A.S. 26AC) placed at either end of the cage.

During ABR and ECoG recording, a ZIF‐clip digital headstage was attached to the implanted electrode, and linked to the recording computer via a TDT Medusa headstage amplifier connected to a TDT System 3 interface. Recordings were collected with lights switched off in the chamber. Online data collection was facilitated by either Brainware (software developed by J. Schnupp, University of Oxford, UK) or custom‐written Matlab scripts, depending on the types of responses being recorded. Recorded ECoG signals were filtered online between 0.5–300 Hz for resting‐state oscillatory activity, 100–5000 Hz for ABRs and 60–300 Hz for all other stimuli. All data were analysed offline with custom‐written Matlab scripts.

### Salicylate and vehicle treatment

Following baseline data collection (see below), we administered sodium salicylate (350 mg/kg; i.p.) dissolved in saline to awake GPs and examined the effects on cortical ECoG activity 2 h later, as well as on suprathreshold ABR responses, in eight of the 13 GPs. This time‐point was selected as previous studies have demonstrated effects on behaviour and neural activity 2 h after salicylate (Guitton *et al*., 2003; Ralli *et al*., 2010; Berger *et al*., 2013). We have also previously demonstrated behavioural effects of salicylate at this dose (Berger *et al*., 2013). Furthermore, in five of these eight GPs, vehicle treatment consisting of saline without salicylate was performed 2 days prior to administering salicylate and all the electrophysiological markers mentioned above were assessed, to determine whether effects seen were simply caused by an intraperitoneal injection, as opposed to direct effects of salicylate.

### ABR stimuli and recording

ABRs were recorded from cerebellar electrodes using custom‐written Matlab scripts. Tones of five different frequencies between 5 and 20 kHz (70 dB SPL, 5 ms duration, 0.1 ms on/off ramp) were presented in a randomized order, the signal was presented with alternating polarity to allow cancellation of any stimulus artefact. A total of 500 repeats for each condition were presented during each session. Offline, signal sweeps with an RMS greater than three absolute deviations above the median were rejected from further analysis and the rest of the data were averaged across repeats. Median absolute deviation (MAD) was used for artefact rejection as it provides a more robust measure of dispersion than standard deviation (Leys *et al*., 2013). The peak‐to‐trough amplitude of the largest ABR response was compared before and 2 h after salicylate administration. The largest ABR response was typically recorded as starting ~ 3 ms after sound presentation. However, ~ 1.5 ms of this delay was accounted for by the time for stimuli to travel the distance between the free‐field speaker and the GP's head. Therefore, the latency between sound presentation at the GP's ears and the start of the largest ABR response prior to salicylate administration was ~ 1.5 ms, consistent with wave I of the subcutaneously recorded ABR (e.g. Dehmel *et al*., 2012). Preliminary data from three GPs, in which we recorded auditory nerve compound action potentials from the round window of the cochlea concurrently with the cerebellar signals, further suggested that the largest deflection in the cerebellar electrode signals corresponded to wave I of the subcutaneously recorded ABR, which is indicative of auditory nerve activity.

### Oscillations and click‐evoked responses

Resting‐state oscillatory activity and click‐evoked responses were recorded using Brainware before and after salicylate administration. Data from four baseline sessions were averaged, with 100 repeats recorded during each session. For resting‐state activity, this comprised 10 s sweeps of recording in silence (1000 s total per session). Data were analysed offline using custom‐written Matlab scripts. Artefact rejection involved including data only with an RMS less than three absolute deviations above the median. Power spectral analysis was performed on every 0.5 s sample of this cleaned data set, resulting in an effective spectral smoothing of 2 Hz, and values were log‐transformed in order to express power in decibels (dB).

Neural activity was recorded in response to short clicks (50 μs), with signals attenuated between 0–40 dB of full output (~ 100 dB SPL RMS), in 10 dB steps, with an interstimulus interval (ISI) of 1500 ms. To compare between recordings during baseline and 2 h after salicylate administration, peak‐to‐trough amplitudes were measured in the 50 ms following the stimulus.

Statistical analysis comparing baseline resting‐state activity with that recorded 2 h after salicylate administration was performed using a cluster‐based permutation test (Maris & Oostenveld, 2007). This test allows for non‐parametric testing of EEG signals and is a more sensitive measure than multiple *t*‐tests with Bonferroni corrections applied. For each frequency, a two‐tailed, one sample *t*‐test was performed. All frequencies were selected for which their *t*‐value exceeded a pre‐designated threshold (uncorrected *P *<* *0.05), and these were then clustered on the basis of spectral adjacency. *t*‐Values within each cluster were summed and this was used as the cluster‐level statistic. Subsequently, the maximum summed cluster was used as a test statistic. Data for each GP were then randomized across conditions (baseline/after salicylate) and this method was repeated for all possible permutations of the data (*n *=* *256). This produced a distribution of maximum cluster *t*‐values to which we could reference the actual data, taking clusters which fell outside the 95% confidence interval of the distribution of *t*‐values as significant.

### Neural gap detection thresholds

Neural gap detection thresholds were determined using the method described in Berger *et al*. (2014). Briefly, auditory stimuli comprised a broadband noise (BBN) burst (duration of 200 ms, on/off ramps of 0.5 ms), followed by a fixed‐length period of silence or ‘gap’ (durations of either 1, 2, 4, 8, 10, 20, 50 or 75 ms) and a final BBN burst (duration of 50 ms, on/off ramps of 2 ms), all presented at 70 dB SPL. Each gap width condition was presented in ascending order (50 repetitions, 1500 ms ISI). Responses were averaged across repetitions, according to each condition. MGDTs were defined as the minimum gap duration where a significant increase in activity could be detected following the onset of the post‐gap stimulus. A significant increase was conservatively determined as any peak response > 5 standard deviations above the mean activity recorded during a 50 ms window in the first 200 ms.

### Behavioural measure of gap detection ability

In a separate group of GPs (*n *=* *5), both behavioural gap detection abilities and neural responses to the same stimuli (GIREP) were measured. The behavioural method used to identify animals experiencing tinnitus in this study is based on a gap detection paradigm devised by Turner *et al*. (2006) in which we measured flexion of the pinna, or the Preyer reflex (Berger *et al*., 2013). The magnitude of the Preyer reflex is calculated as pinna displacement under different acoustic conditions, and these measurements are used to quantify gap‐induced pre‐pulse inhibition (PPI) of the reflex. Following interventions that are known to cause tinnitus, PPI is compromised. This method is described elsewhere (Berger *et al*., 2013; Coomber *et al*., 2014).

### Baseline behavioural testing

Baseline PPI of the Preyer reflex was measured in each GP over a 2‐week period (minimum of three and a maximum of six testing sessions). Startling stimuli (BBN bursts of 20 ms; rise/fall time of 1 ms) and continuous background noise conditions (either BBN or 2 kHz wide narrow‐band noise centred at 5, 9, 13 or 17 kHz) were used, as described previously (Berger *et al*., 2013, 2014; Coomber *et al*., 2014). A gap duration of 50 ms was used to elicit gap‐induced PPI, consistent with that used by others (e.g. Turner *et al*., 2006, 2012; Turner & Parrish, 2008). Sound levels for the behavioural test were determined for each GP as described in our previous work. Briefly, optimal sound levels of startling stimuli (95, 100, or 105 dB SPL) and background carrier (55, 60 or 70 dB SPL) were chosen to maximize baseline PPI for each animal (sound level‐dependency test; see Berger *et al*., 2013).

After baseline data collection, GPs that exhibited significant PPI in all background sound conditions were chronically implanted, their behavioural gap detection ability was reassessed following recovery and neural gap detection abilities determined, before administering sodium salicylate and recording the same responses 2 h later. Oscillatory activity, click‐evoked responses and MGDTs were not recorded for these GPs due to time constraints.

### Gap‐induced reductions of evoked responses (GIREP)

The same stimuli used in the behavioural test were also presented while recording neural activity, to allow for direct comparison of behaviour and neural activity. This included using the same sound levels and background frequencies, with the same number of repetitions (10 gap/no‐gap conditions for each frequency. Peak‐to‐trough amplitudes of ECoG signals in the 50 ms following the startling stimulus, averaged across repeats, were calculated for both no‐gap and gap conditions. In a similar manner to the behavioural test, a percentage difference between ‘gap’ and ‘no‐gap’ data were then calculated and GIREP was expressed as a percentage decrease in peak‐to‐trough amplitude when a gap was presented, compared with the ‘no‐gap’ condition. Data from all animals were pooled and the effects of salicylate on the GIREP were assessed statistically for each background noise condition with a two‐way anova and Bonferroni *post hoc* test.

## Results

ECoG data from the two sides were grouped prior to analysis, as hemispheric differences were not expected as a result of an intraperitoneal injection and no clear differences were observed.

### Changes in ABRs following salicylate

**Figure 2:**
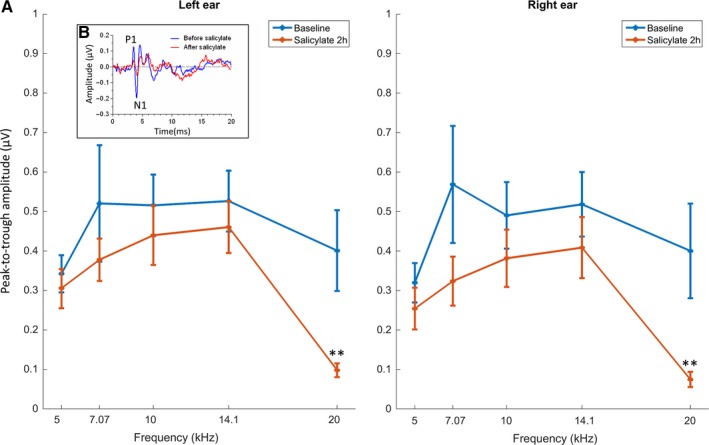
(A) Peak‐to‐trough amplitudes of wave I ABRs recorded from the cerebellar electrodes, for all 5 frequencies from left ear (left panel) and right ear (right panel). Data averaged across GPs (*n *=* *5) and shown for before vs. 2 h after salicylate (**P <* *0.01). Error bars indicate SEM. B (inset): Example responses from the cerebellar electrodes from one GP in response to 20 kHz tones, before vs. after salicylate administration. P1 and N1 of the largest wave (ABR wave I) are labelled. GPs, guinea pigs. [Colour figure can be viewed at wileyonlinelibrary.com]

Suprathreshold (70 dB SPL) ABRs were recorded before and 2 h after salicylate administration. A total of five different pure tone frequencies, each one half an octave apart from the next, were tested: 5, 7.07, 10, 14.1 and 20 kHz. An example ABR from one GP before and after salicylate administration is shown in Fig. 2B (inset). Figure 2A shows the peak‐to‐trough amplitudes of the largest wave (wave I) from the cerebellar electrodes before and after salicylate administration for the five different frequencies, averaged across GPs. Overall, there was a significant effect of treatment on ABR amplitudes for both the left ear (*F*_1,19_ = 11.78, *P *=* *0.0028) and the right ear (*F*_1,20_ = 17.90, *P *=* *0.0004), as identified using a two‐way repeated measures anova. Bonferroni *post hoc* analysis revealed that there was a significant reduction in amplitude at 20 kHz following salicylate administration (left ear: *t* = 3.805, *P *<* *0.01; right ear: *t* = 3.605, *P *<* *0.01). This indicates that there was reduced sensitivity in suprathreshold hearing for both ears at this frequency. The amplitudes of the responses at all other frequencies measured were slightly reduced, but did not reach statistical significance.

Furthermore, there was also a significant increase in wave I P1 latency at 20 kHz for left and right ears across GPs (left ear: *t* = 4.153, *P *<* *0.01, right ear: *t* = 3.678, *P *<* *0.01), with mean increases [± standard error of the mean (SEM)] of 0.13 ms (± 0.04 ms) and 0.12 ms (± 0.03 ms) respectively. Significant increases in latency were also evident for N1 of the wave I (left ear: *t* = 4.613, *P *<* *0.01; right ear: *t* = 3.257, *P *<* *0.01), with mean increases (± SEM) of 0.16 ms (± 0.03 ms) and 0.12 ms (± 0.03 ms). There were no significant changes in latency at any other frequencies for P1 or N1 measured from either ears.

### Changes in oscillatory activity following salicylate

**Figure 3:**
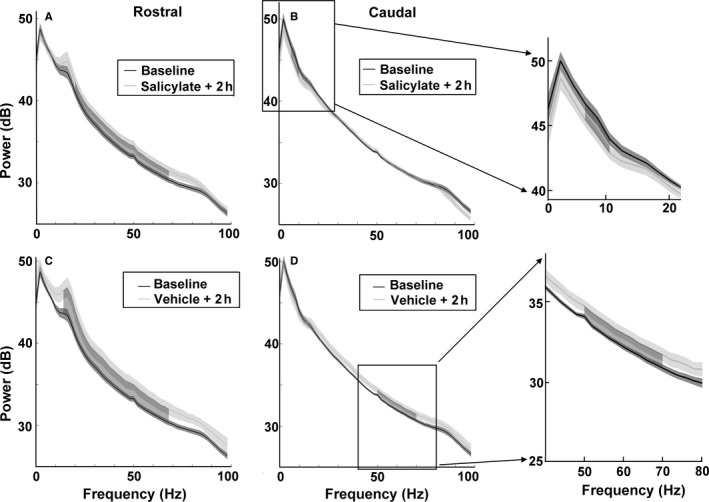
Power spectra of resting‐state activity from rostral AC (A) and caudal AC (B). Black lines and shading represent mean spectra (± SEM) of baseline recordings across GPs (*n *=* *8), while light grey lines and shading represent mean spectra (± SEM) of recordings 2 h after salicylate administration. Data are also shown following vehicle administration (C and D). Dark shading between lines highlights significant differences, determined using a cluster‐based permutation test (Maris & Oostenveld, 2007). GPs, guinea pigs.

Fast Fourier transforms were applied to resting‐state ECoG data collected during silence to produce power spectra. Figure 3 shows the mean power spectra for eight GPs, collected before and 2 h following salicylate administration. Statistical significance was identified using the cluster‐based permutation test, with an alpha level of *P *<* *0.05. There was a significant reduction in power following salicylate (compared to baseline) between 6–10 Hz on the caudal cortical electrodes (Fig. 3B), while a significant increase in power was evident between 20–68 Hz on the rostral cortical electrodes (Fig. 3A). However, an increase in power on the rostral electrodes was also evident (Fig. 3C) 2 h following vehicle treatment at similar frequencies (14–68 Hz), suggesting that this effect was a result of the stress associated with the intraperitoneal injection rather than with salicylate *per sé*. An increase in power was also evident on the caudal electrodes between 50–70 Hz following vehicle treatment (Fig. 3D). In summary, the reduction in power between 6–10 Hz on the caudal electrodes appeared to be the only effect on oscillatory activity unique to salicylate administration.

### Changes in click‐evoked responses following salicylate

**Figure 4:**
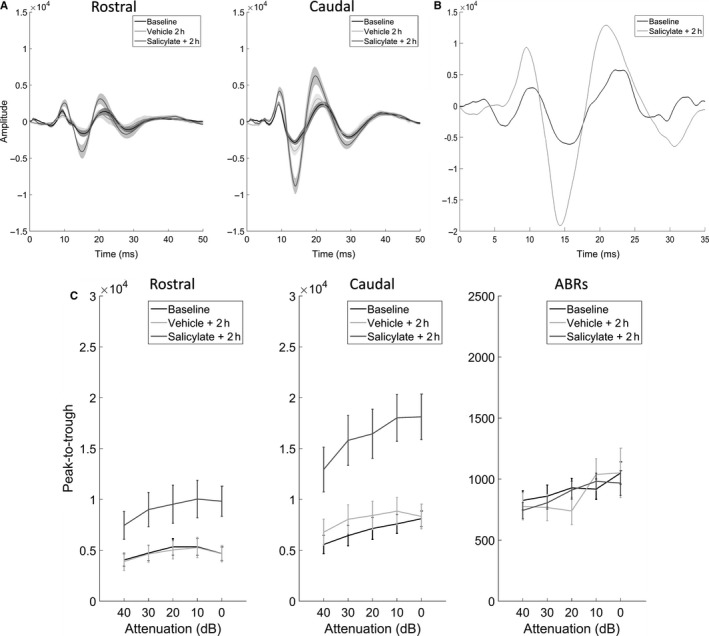
Click‐evoked potentials. (A) Mean responses across guinea pigs (GPs) (± SEM;* n *=* *8) from rostral AC and caudal AC, recorded during baseline (black), 2 h after vehicle (light grey) and 2 h after salicylate (dark grey). (B) An example trace of a single click‐evoked response before vs. 2 h after salicylate. (C) Mean peak‐to‐trough amplitudes of click‐evoked responses across GPs (± SEM) for all attenuations recorded during baseline, 2 h after vehicle and 2 h after salicylate from rostral AC (left panel), caudal AC (middle panel) and ABRs (right panel).

Figure 4A shows responses to short clicks (50 μs), averaged across eight GPs and recorded with a signal attenuation of 0 dB SPL (approximately 100 dB SPL RMS) before and 2 h after salicylate administration, as well as 2 h after vehicle administration. Clear increases in click‐evoked potentials were evident at all sound levels 2 h after salicylate administration for both rostral and caudal AC (Fig. 4C, left and middle panels). Increases ranged from 75 to 106% for rostral AC and 134–149% for caudal AC, depending on the sound level presented. These increases were clear to the extent that they were often apparent in a single recording sweep for each GP (Fig. 4B). There were no clear changes in the amplitudes of click‐evoked responses on the ABR channels (Fig. 4C, right panel). Furthermore, no clear differences in click‐evoked potentials were evident 2 h following vehicle administration when compared to baseline recordings.

### Neural gap detection thresholds

**Figure 5:**
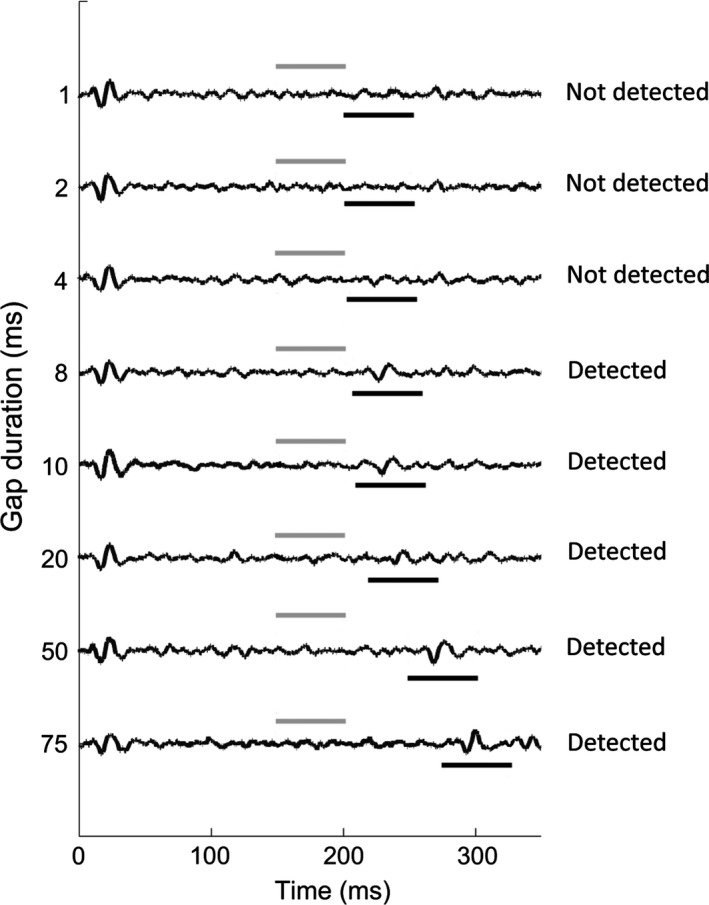
An example of how neural gap detection thresholds were determined. Gaps were considered detected if there was a significant increase in activity during presentation of the second 50 ms stimulus (indicated by the black lines below the traces) compared to a 50 ms period during the first 200 ms stimulus (indicated by the grey lines above the traces). Minimum gap detection thresholds were defined as the lowest gap duration that elicited this difference.

Example responses used to determine MGDTs are shown in Fig. 5. MGDT results before and 2 h after salicylate administration for each GP are shown in Table 1. On the rostral AC electrodes, the median MGDT was 8 ms (± 1 MAD) during baseline recordings. Two hours following salicylate, the median had increased to 15 ms (± 12.5 MAD), although there was variability in MGDTs; two GPs actually improved in their MGDTs, two showed minor decreases in threshold and four showed more extensive decreases. However, only one GP of these four had an MGDT greater than 50 ms, the gap duration used for the behavioural test. On the caudal AC electrodes, the median MGDT was 4 ms (± 2 MAD) during baseline and then increased to 8 (± 4 MAD) following salicylate. One GP showed a reduction in MGDT, two showed no change, three had minor increases in MGDTs and two had more extensive increases. Again, there was only one GP that had an MGDT of greater than 50 ms following salicylate; this was the same GP that had an MGDT of 75 ms on the rostral AC electrodes (GP3 in Table 1). These results indicate that while there were some changes in MGDTs, gaps of 50 ms duration, the duration used in the behavioural test, were detectable by the majority of GPs following salicylate administration.

**Table 1:** Minimum gap detection thresholds (MGDTs) (in ms) before vs. 2 h after salicylate for each individual GP, recorded from rostral AC and caudal AC. Median MGDTs [± median absolute deviations (MAD)] are shown at the bottom of each column

GP	Rostral		Caudal	
	Before	After	Before	After
1	8	50	4	8
2	8	10	8	50
3	8	75	8	75
4	10	50	4	4
5	4	20	2	8
6	8	4	4	1
7	4	1	8	8
8	2	4	2	4
Medians	8	15	4	8
MAD	1	12.5	2	4

### Responses to behavioural gap detection stimuli

**Figure 6:**
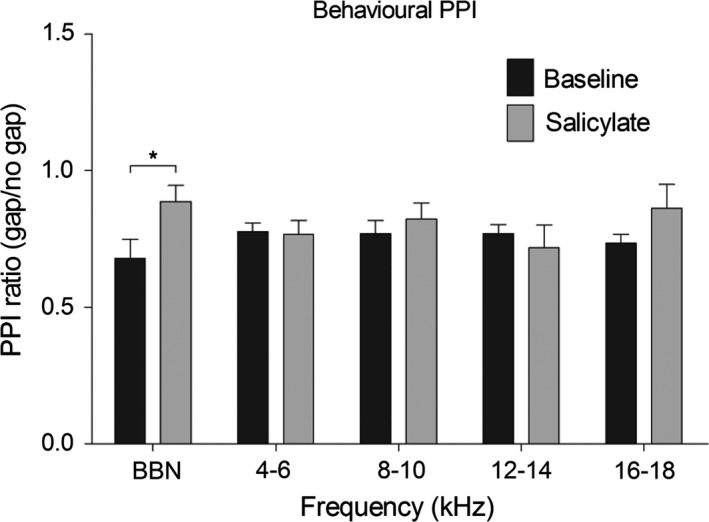
Behavioural gap‐induced PPI data recorded for five different background frequencies before (black) and 2 h after (grey) salicylate administration. Data are shown as gap responses/no‐gap responses and are averaged across GPs (*n *=* *5). A value of 1 would indicate that the gap was not detected at all, whereas a value lower than one would suggest that the gap was inhibiting the response. Error bars represent SEM (*P <* *0.05). GPs, guinea pigs; PPI, pre‐pulse inhibition.

In a separate group of GPs (*n *=* *5), gap detection was measured behaviourally using the method described in Berger *et al*. (2013). These data are summarized in Fig. 6. A two‐way repeated measures anova with Bonferroni *post hoc* test was used to statistically compare data for all GPs collected after salicylate administration with baseline recordings. There was a significant effect of treatment on behavioural performance across GPs (*F*_1,19 _= 4.45, *P *=* *0.0484), whereby gap‐induced PPI was significantly smaller 2 h after salicylate. *Post hoc* analysis revealed that this effect was restricted to the BBN background carrier (*t *= 3.805, *P *<* *0.01). These data are consistent with the results from Berger *et al*. (2013) also collected 2 h after salicylate administration. Furthermore, behavioural startle response amplitudes across GPs to ‘no‐gap’ stimuli were significantly enhanced 2 h following salicylate (*P *=* *0.0002; Wilcoxon Signed‐Rank test), from a mean of 4.98 mm (± 0.72 mm SEM) to 7.93 mm (± 0.95 mm SEM). This was also consistent with our previous results following salicylate administration.

**Figure 7:**
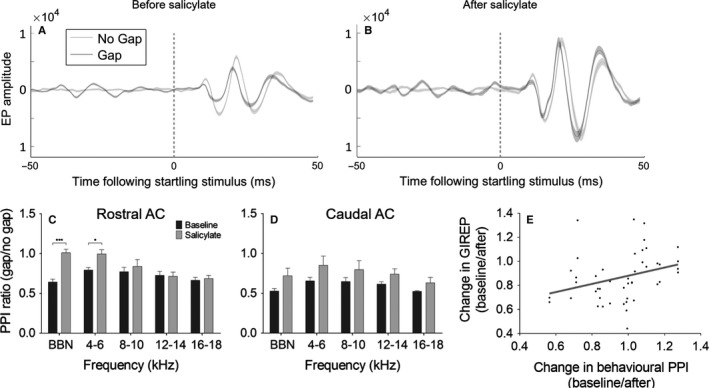
Gap‐induced reductions of evoked potentials (GIREP). (A,B) Example responses recorded from AC electrodes for 1 GP to a BBN startling stimulus embedded in continuous background noise with no‐gap preceding (light grey), compared to responses with a 50 ms gap preceding the startling stimulus (dark grey) are shown before (A) and 2 h after (B) salicylate administration. (C, D) Mean ratios of gap responses/no‐gap responses recorded from rostral AC (C) and caudal AC (D) before and after salicylate administration for the same five frequencies as presented in the behavioural test. As with the behavioural data, a value of 1 would indicate that the gap was not having an effect on evoked responses, whereas a value lower than one would suggest that the gap was inhibiting the evoked response. Error bars indicate SEM (***P <* *0.001; *P <* *0.05). (E) Linear regression analysis for change in GIREP vs. change in behavioural PPI in the same GPs following salicylate administration. A value of 1 would indicate no change in GIREP or behavioural PPI, whereas a lower value would suggest a deficit in either measure. GPs, guinea pigs; BBN, broadband noise; PPI, pre‐pulse inhibition.

The same stimuli used in the behavioural test were also presented while recording ECoG from the same group of GPs that had been behaviourally tested. Figure 7A shows example AC responses to a BBN stimulus with and without a preceding gap in background noise. Prior to salicylate administration, we were able to successfully inhibit evoked potentials by presenting a gap prior to the startle‐eliciting stimulus (GIREP), on both rostral and caudal AC electrodes for all GPs (as in the example in Fig. 7A). Following salicylate administration, there were frequency‐specific deficits in GIREP across GPs on the rostral electrodes, an example of which is shown in Fig. 7B. Overall, for the rostral AC electrodes (Fig. 7C), there was a significant effect of salicylate treatment on GIREP (*F*_1,19 _= 22.62, *P *=* *0.0001), a significant difference between frequencies (*F*_4,19 _= 4.28, *P *=* *0.0123) and a significant interaction between the two factors (*F*_4,19 _= 6.08, *P *=* *0.0025). This effect was strongest for BBN, where there was a significant deficit in GIREP (*t* = 5.554, *P *<* *0.001), which was consistent with the BBN deficit observed in behavioural responses in the same GPs. A significant, but smaller deficit was also evident in GIREP at 4–6 kHz (*t* = 3.386, *P *<* *0.05).

On the caudal AC electrodes (Fig. 7D), there was a significant effect of salicylate treatment overall (*F*_1,19_ **=* *14.75, *P *=* *0.0011), but no effect of frequency (*F*_4,19_ **=* *1.62, *P *=* *0.2100) nor interaction (*F*_4,19_ **=* *0.20, *P *=* *0.9355), indicating that while there was a general worsening in GIREP, this was not restricted to a particular frequency on the caudal AC electrodes. Linear regression analysis demonstrated that there was a significant positive correlation between the change in GIREP across electrodes and change in behavioural PPI in the same GPs following salicylate (*r* ^2^ **=* *0.10, *P *<* *0.05), that is, GPs with a greater deficit in GIREP generally exhibited a greater deficit in behavioural PPI (Fig. 7E). There was also a significant positive correlation between GIREP and PPI in general, indicating that a greater GIREP (gap/no‐gap) ratio correlated with a greater behavioural PPI ratio (gap/no‐gap) in the same GPs (*r* ^2^ **=* *0.06, *p *<* *0.05).

**Figure 8:**
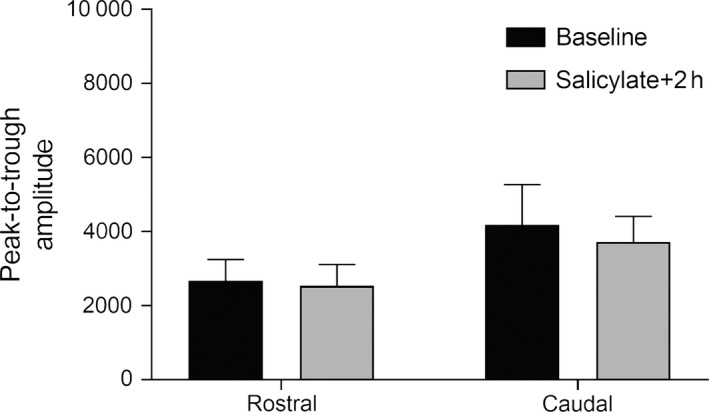
Peak‐to‐trough amplitudes of gap termination responses for broadband noise gap‐induced reductions of evoked potentials stimuli, occurring prior to the startling stimulus, for rostral and caudal electrodes before vs. after salicylate administration.

In summary, the deficits in GIREP observed in data recorded from the rostral AC electrodes are aligned with those observed behaviourally. We also analysed offset responses to gaps (gap termination responses) for the GIREP stimuli before and after salicylate, which were evident in response to BBN stimuli prior to the startling stimulus (occurring in the −50 to 0 ms window in Fig. 7A and B). Despite a significant deficit in GIREP in the rostral electrodes for BBN, a two‐tailed paired *t*‐test indicated that there were no significant changes in the peak‐to‐trough amplitudes of gap termination responses to the same stimuli (*t *=* *0.55, *P *=* *0.61), highlighting that the deficits in GIREP could not be explained by deficits in gap termination responses (Fig. 8). There were also no deficits in BBN gap termination responses for the caudal electrodes (*t *=* *0.65, *P *=* *0.55).

## Discussion

A variety of changes in neural activity in awake animals were observed following salicylate administration. There were reductions in peripheral hearing sensitivity, although large changes were restricted to the highest frequency tested (20 kHz), changes in cortical oscillations unique to salicylate, increases in cortical auditory evoked potentials (despite reduced hearing sensitivity) and generally minor changes in gap detection thresholds. Furthermore, to our knowledge, this is the first demonstration of gap‐induced reductions of auditory cortex evoked potentials in animals (as opposed to commonly examined gap termination responses), as well as the first study to demonstrate such deficits following salicylate administration. This may be clinically relevant because it has been suggested that cortical recording of this sort might be the basis of an objective test for tinnitus (Suh *et al*., 2013).

### Cortical oscillatory activity is changed following sodium salicylate administration

Significant reductions in oscillatory power specific to salicylate administration (as opposed to vehicle injection) were restricted to the 6–10 Hz region on the caudal cortical electrodes, putatively over the dorsocaudal area. These frequencies encompass the lower end of alpha band oscillations, which have a bandwidth of 8–12 Hz and play a role in inhibitory function (Klimesch, 2012). Several studies have demonstrated reductions in alpha band activity power in patients with noise‐induced tinnitus (Weisz *et al*., 2005; Adamchic *et al*., 2014; Schlee *et al*., 2014), although others have failed to find such changes (e.g. Ashton *et al*., 2007; Adjamian *et al*., 2012). Furthermore, Weisz *et al*. (2005) and Adjamian *et al*. (2012) suggested that increases in delta band activity may also be important in tinnitus generation, something which is not evident in our data.

Reductions in alpha activity form part of the basis for the idea of thalamocortical dysrhythmia (Llinas *et al*., 1999; De Ridder *et al*., 2015). Under this hypothesis, tinnitus is characterized by a reduction in alpha activity in conjunction with an increase in gamma activity. Our data do not fully support this idea being applicable to salicylate‐induced tinnitus, however, as although we found an increase in gamma activity in rostral AC, this was also evident following vehicle treatment, whereas the reduction in 6–10 Hz activity was unique to salicylate administration. This suggests that the change in gamma may have been related to stress, which is known to be caused by IP injections (Meijer *et al*., 2006). There is little published work on the effects of stress on cortical oscillations in the auditory cortex directly. However, acute stress is known to affect the intensity of paradoxical sleep in rodents (Meerlo *et al*., 1997) and two periods of paradoxical sleep occur on average every hour throughout the day even in head‐restrained GPs (Escudero & Vidal, 1996). Paradoxical sleep is associated with increased gamma band oscillations and these may have been picked up in our recordings. Furthermore, fear conditioning enhances gamma band oscillatory activity in auditory cortex (Headley & Weinberger, 2013). Although vehicle injections also increased gamma band activity, it is still possible that a combination of reduced inhibitory alpha activity in the dorsocaudal area and an increase in gamma band activity in primary AC could contribute to the tinnitus percept.

It is generally assumed that high doses of salicylate reliably produce the symptom of tinnitus, but we did not behaviourally test the same animals from which we recorded oscillatory ECoG activity. However, we demonstrated tinnitus‐like behavioural deficits following the same dose of salicylate in a separate group of GPs, consistent with our previous study (Berger *et al*., 2013). Behavioural data were collected 2 h after administration, as were ECoG data from GPs where oscillatory activity was recorded, thereby suggesting that the GPs that were not behaviourally tested would also have been likely to have experienced tinnitus at this time‐point. However, differences in tinnitus induction (salicylate vs. noise exposure) could explain inconsistencies with human studies, and further research is required to determine changes in ECoG activity following noise exposure that may relate to tinnitus.

### Auditory evoked responses are increased despite reduced peripheral sensitivity

There were clear enhancements in evoked responses at both rostral and caudal cortical electrodes 2 h following salicylate. These were present despite a reduction in the amplitude of wave I brainstem responses to 20 kHz tones and no change in the cerebellar ABR to clicks, indicating that high frequency auditory nerve activity was reduced, likely due to the ototoxic effects of salicylate (see Cazals, 2000 for a review). These results are consistent with the effects of salicylate shown previously (Sun *et al*., 2009; Fang *et al*., 2016) and could suggest a compensatory mechanism in line with the central gain theory of tinnitus (Schaette & Kempter, 2006; Schaette & McAlpine, 2011), in which a reduction in peripheral sensitivity initiates homeostatic mechanisms that increase central auditory activity. Furthermore, increased evoked activity could be an indication of hyperacusis, an oversensitivity to sound that is commonly comorbid with tinnitus (Baguley, 2003). Indeed, we also observed enhanced startle amplitudes following sodium salicylate, a behavioural measure which has previously been suggested as a correlate of hyperacusis (e.g. Chen *et al*., 2013). Direct effects of sodium salicylate on the brain could be responsible for this sound‐evoked enhancement and may not parallel changes occurring following noise exposure (Auerbach *et al*., 2014), although Norena *et al*. (2010) demonstrated similar effects on evoked potentials for both noise exposure and sodium salicylate in awake GPs. This therefore suggests that these two tinnitus inducers may cause gain enhancement in a similar manner, despite different timescales.

It is interesting that increased evoked activity was evident on both the rostral and caudal electrodes following salicylate administration, but that reductions in alpha band activity were only evident on the caudal electrodes. This suggests that there may be different pathways involved in hyperacusis (increased evoked responses) compared with tinnitus (changes in oscillatory activity evident in silence). Indeed, disparate mechanisms between the two often co‐morbid phenomena have previously been suggested (Knipper *et al*., 2013).

### Alterations in gap detection ability following salicylate administration

Deficits in behavioural gap detection performance following either salicylate administration or noise exposure are often taken as a correlate of tinnitus in animals (Turner *et al*., 2006, 2012; Turner & Parrish, 2008; Longenecker & Galazyuk, 2011; Dehmel *et al*., 2012; Berger *et al*., 2013; Chen *et al*., 2013; Coomber *et al*., 2014), although doubt has been cast as to the efficacy of this model in detecting tinnitus (Campolo *et al*., 2013; Fournier & Hebert, 2013; Lobarinas *et al*., 2013). Here, we found that there were some increases in neural gap detection thresholds (in response to BBN) 2 h following sodium salicylate administration. This was consistent with the results of Berger *et al*. (2014) in the inferior colliculus following noise exposure, as well as Deng *et al*. (2010), who demonstrated increases in auditory cortex MGDTs following salicylate administration in rats. However, there was only one GP with minimum neural gap detection thresholds of greater than 50 ms, thereby suggesting that, for the majority of GPs, their temporal acuity was not affected to an extent that they would no longer be able to perform the behavioural task. This was true also in the Deng *et al*. (2010) study, where auditory cortex MGDTs were still far below 50 ms following salicylate.

Contrastingly, behavioural responses to a BBN background carrier were significantly altered 2 h following sodium salicylate administration (consistent with the results of Berger *et al*., 2013). Interestingly, the same was true for neural responses to the behavioural gap detection stimuli. When gap termination responses were analysed to these same stimuli, which are usually used for determining MGDTs, there were no differences in amplitudes following salicylate. These data suggest that there are separate mechanisms behind the two different tasks; that is, the reduction of either evoked neural responses to a startling stimulus in auditory cortex or behavioural startle responses by a preceding gap may not directly relate to absolute gap detection thresholds in auditory cortex. The gap‐induced PPI circuit has been attributed to the brainstem (Lowe & Walton, 2015), but is thought to be subject to descending modulation by structures such as the cortex and amygdala (Bosch & Schmid, 2008). Thus, decorticate animals show impairments in GPIAS for gaps of < 50 ms duration (Ison *et al*., 1991; Threlkeld *et al*., 2008), suggesting that the cortex still plays a role in gap‐induced PPI at shorter gap durations. Interestingly, Weible *et al*. (2014) found that suppressing cortical neural activity using optogenetics following a gap could attenuate subsequent behavioural startle responses for gaps with durations of ≤ 25 ms, but not for 50 ms gaps (the duration used here), which further suggests that cortical involvement is important only for shorter durations. Therefore, the effect on gap‐induced reductions of cortical evoked potentials that we have observed here may simply be a reflection of processing further down the auditory system. On the other hand, responses to the neural gap detection threshold stimuli, whereby onset responses to noise following a gap (i.e. offset responses to the gap or gap termination responses) are examined, may reflect processes occurring above the level of the brainstem.

An alternative explanation is that the small changes in MGDTs we observed following salicylate administration may have been sufficient to have caused significant deficits in behavioural gap detection ability. Under this scenario, behavioural deficits following tinnitus induction would reflect impaired acuity, as opposed to the original hypothesis of tinnitus filling the gap (Turner *et al*., 2006).

Regardless of the mechanism underlying the disparity between the two measures of gap detection ability, an important point regarding the relationship between behavioural testing in animals and gap detection in humans with tinnitus is highlighted. Namely, simply showing that there are no clear differences in psychophysical gap detection in humans with tinnitus (or animals for that matter), as demonstrated by Campolo *et al*. (2013), Boyen *et al*. (2015) and Radziwon *et al*. (2015), may not be sufficient to tell us whether or not the behavioural test can determine whether animals are experiencing tinnitus, It is likely more important to demonstrate deficits in gap‐induced PPI of a startle response in humans to support the idea that this test is a suitable objective test for tinnitus in animals. The data above suggest that there are likely important differences between gap‐induced PPI of a reflex response and absolute gap detection thresholds.

An important confound of the behavioural GPIAS test is that interventions that are known to induce tinnitus may also cause hearing loss. Lobarinas *et al*. (2013) demonstrated that simulating hearing loss in animals can result in false positives in the GPIAS test, as startle amplitudes are reduced (for a review of hearing loss and the GPIAS test, see Galazyuk & Hebert, 2015). However, although we found that hearing loss was present in GPs in this study, startle amplitudes were in fact increased following salicylate administration, consistent with the results of other studies (Sun *et al*., 2009; Chen *et al*., 2013). Furthermore, the clearest deficit in behaviour was evident for BBN stimuli, and we found that there were no clear changes in ABR amplitudes for BBN (click) stimuli. Therefore, this confound should not have affected interpretation of the data presented here, although this is nonetheless an important consideration when attempting to induce tinnitus with noise exposure, which may cause a significant decrease in startle response amplitudes.

Neural activity recorded here following salicylate administration suggests that there are fundamental changes that could give rise to the presence of tinnitus. Importantly, there is evidence that indicates hyperacusis‐like effects are present and measurable in animals – namely, increases in evoked auditory potentials and increases in startle response amplitudes. Hyperacusis is a common comorbidity in patients with tinnitus, with one study suggesting that it is present in up to 86% of cases (Anari *et al*., 1999). Despite this, it is an often overlooked issue in research (Moller *et al*., 2015). Further understanding the mechanisms that give rise to such a phenomenon may elucidate changes that also underlie the presence of tinnitus.

## Conflict of interests

The authors declare no competing financial interests.
